# Anxiety-Related Modulation of Early Neural Responses to Task-Irrelevant Emotional Faces

**DOI:** 10.3390/brainsci16010026

**Published:** 2025-12-25

**Authors:** Eligiusz Wronka

**Affiliations:** Institute of Psychology, Jagiellonian University, 31-007 Kraków, Poland; eligiusz.wronka@uj.edu.pl

**Keywords:** anxiety, emotional expression, face inversion, event-related potentials

## Abstract

**Objectives**: The purpose of the study was to test the hypothesis that high anxiety is associated with biased processing of threat-related stimuli and that anxious individuals may be particularly sensitive to facial expressions of fear or anger. In addition, these effects may result from a specific pattern occurring in the early stages of visual information processing. **Methods**: Event-Related Potentials (ERPs) were recorded in response to task-irrelevant pictures of faces presented in either an upright or inverted position in two groups differing in trait anxiety, as assessed by scores on the Spielberger Trait Anxiety Inventory (STAI). Behavioural responses and ERP activity were also recorded in response to simple neutral visual stimuli presented during exposure to the facial stimuli, which served as probe-targets. **Results**: A typical Face Inversion Effect was observed, characterised by longer latencies and greater amplitudes of the early P1 and N170 ERP components. Differences between low- and high-anxious individuals emerged at parieto-occipital sites within the time window of the early P1 component. The later stage of face processing, indexed by the N170 component, was not affected by the level of trait anxiety. **Conclusions**: The results of this experiment indicate that anxiety level modulates the initial stages of information processing, as reflected in the P1 component. This may be associated with anxiety-related differences in the involuntary processing of face detection of emotional expression. Consequently, a greater attentional engagement appears to occur in highly anxious individuals, leading to delayed behavioural responses to concurrently presented neutral stimuli.

## 1. Introduction

Human faces are highly salient stimuli that convey significant nonverbal signals, and they are regarded as the most direct indicator of current affective dispositions and attitudes, both positive and negative. Owing to their biological and social significance, information about emotional states derived from faces must be processed rapidly in order to support immediate behavioural regulation. 

There is a large body of evidence suggesting the existence of a distinct and highly specialised brain module critically involved in face processing [1,2]. The regions generally engaged in the processing of faces are also strongly activated during the processing of facial emotions. Early perceptual analysis occurs in the inferior occipital cortex [3] and in the lateral fusiform gyrus [4] for invariant aspects of faces that support the determination of face identity [2,5,6], whereas the superior temporal sulcus is involved in processing changeable aspects of faces, such as facial expression and eye and mouth movements [1]. These regions of the visual cortex are also strongly interconnected with numerous other brain areas involved in evaluating signals relevant to social functioning. Specifically, it has been proposed that the amygdala and orbitofrontal cortex mediate a rapid, preattentive evaluation of the emotional and motivational significance of facial expression [7], while the anterior cingulate, prefrontal cortex, and somatosensory areas contribute to forming conscious representations of facial emotional expressions [8].

Research on brain structures involved in the perception and analysis of faces and their emotional significance is conducted using several complementary methods, including functional brain imaging, neuropsychological assessment of focal brain lesions, and electrophysiological recordings. The latter have enabled the identification of a highly specific indicator of face processing in the form of the N170 component of event-related potentials (ERPs). The N170 typically appears 140–200 ms after stimulus onset at occipito-temporal sites and has greater amplitude in response to faces compared with other visual stimuli [9,10,11]. Furthermore, higher amplitude and longer latency of the N170 are consistently observed in response to inverted faces compared with upright faces, an effect not reported for other categories of visual stimuli [10,11,12]. There is also a growing body of evidence indicating that the amplitude of the N170 is sensitive to face familiarity (see [13] for a review).

It should also be noted that the findings of numerous studies indicate that the N170 component varies as a function of emotional expression. However, this effect depends on several factors, including the type of task performed by participants. Some early studies failed to demonstrate any influence of emotional expression on the N170 [14,15,16]. Nevertheless, a substantial number of studies have reported modulation of the N170 component by the emotional expression of the presented face [17,18,19,20,21,22,23]. The amplitude of the N170 elicited by emotional faces has been found to be greater than that elicited by neutral faces (see also [24] for review).

The variation in the occipital P1 component, recorded approximately 100 ms after stimulus onset, has been examined far less extensively. Nonetheless, in this case too, a face inversion effect has been observed in some studies [25,26]. Evidence for effects of facial expression on P1 is mixed, with some studies reporting such modulation and others not (see [27] for a review). It has recently been suggested that the intensity of facial emotions may be a crucial factor modulating this early facial expression effect [28].

In line with this, it is generally accepted that measurement of the P1 and N170 components enables the characterisation of the temporal course and intensity of brain activity associated with the early stages of visual processing, including the processing of human faces. However, the patterns of this activity are influenced not only by the content of the presented stimuli (e.g., emotional expression) and the manner in which they are displayed (e.g., upright or inverted), but also by stable individual characteristics of the study participants. One such factor is the level of trait anxiety.

It has been proposed that the cognitive system of individuals with higher levels of anxiety may be distinctively sensitive and may bias the processing of threat-related stimuli relative to neutral ones. Numerous findings demonstrate that anxious individuals exhibit an attentional bias towards threat-related stimuli, an effect that is far less consistently observed in non-anxious individuals [29,30].

Several theoretical models have been developed to explain the attentional bias towards threat in the context of anxiety (see [31] for a review). However, the precise mechanisms underlying the attentional biases observed among anxious individuals remain to be fully clarified. One contributing factor may be the considerable variety of experimental paradigms used in research and the broad diversity of stimuli employed. Studies also compare markedly different groups of participants (e.g., clinical groups with anxiety disorders, individuals with specific anxiety disorders, and subclinical groups with elevated anxiety levels).

A number of differences in the processing of threat-related stimuli have been identified as characteristic of individuals with higher levels of anxiety when compared with low-anxiety groups. These include stronger attentional facilitation (i.e., threat stimuli are detected more rapidly than non-threat stimuli) [32], greater difficulty in disengaging attention (i.e., attention is more difficult to shift away from a threat stimulus than from a neutral stimulus) [33], and less efficient attentional avoidance (i.e., diverting attention towards locations opposite to the threat) [34,35,36]. It has also been proposed that enhanced amygdala activity constitutes a neural mechanism that may mediate anxiety-related hypervigilance for threat [37]. Consequently, individuals with varying levels of anxiety may exhibit differing patterns of brain activity in response to stimuli associated with threat. It can further be assumed that when faces expressing different emotional states are presented, such effects should be most pronounced for expressions of fear or anger.

However, threat detection in faces is understood to involve several stages of information processing, which can be divided into early processes, such as stimulus encoding, and later, more strategic processes reflecting sustained motivated attention and stimulus interpretation. Specifically, it is widely accepted that early, involuntary stages of processing are reflected in the P1 or N170 components, whereas later stages can be assessed by measuring less face-specific components such as the LPP [38,39]. Previous research has demonstrated anxiety-related differences in the early ERP component P1 during the processing of threatening facial expressions, although similar findings have not been reported for the N170.

Anxious participants have been shown to exhibit larger P1 amplitudes in response to fearful facial expressions compared with non-anxious participants [40]. Similar effects have been observed for expressions of anger [41] and for emotional facial expressions more broadly [42]. Anxiety-related enhancement of P1 amplitude has also been recorded when a neutral face is presented in the context of threatening information [43]. These results may suggest that individuals with high anxiety show an early increase in activation of visual cortex regions in response to threat-related stimuli, including human faces. This effect appears to occur at a processing stage that precedes the conscious recognition of the perceived stimulus.

However, a number of studies have failed to confirm this effect, e.g., [44,45], and some have even reported smaller P1 amplitudes in high-anxiety groups [46,47]. It should be emphasised that substantial differences exist in the experimental procedures employed across these studies. For instance, no effect of anxiety level on P1 was observed when participants performed a relatively demanding spatial-attention task [44], or when neutral—but not emotional—faces were aversively conditioned [45]. Smaller P1 amplitudes have also been recorded in high-anxious individuals when fearful faces were presented under conditions that prevented their conscious perception [47]. Another unresolved issue concerns whether the anxiety-related enhancement of P1 amplitude occurs exclusively in response to threat-related expressions or whether it may also be elicited by other facial expressions.

The present study had three aims. First, to replicate the effect of high anxiety on P1 amplitude in a relatively simple perceptual task that does not engage spatial attention. Second, to test the hypothesis that this effect is restricted to threat-related expressions. Third, to examine the impact of face inversion—which disrupts face processing—on the relationship between anxiety level and P1 amplitude.

Participants were presented with task-irrelevant pictures of faces displaying four different emotional expressions in the central visual field. In half of the trials, the faces were inverted. Participants were instructed to detect and respond to a briefly presented neutral visual stimulus appearing in the centre of the picture, which served as a probe-target.

It was hypothesised that individuals with higher anxiety levels would perform more poorly (i.e., exhibit longer RTs) than those with lower anxiety levels, particularly on trials involving threat-related expressions. This would stem from stronger involuntary engagement in processing expressive faces and from difficulties disengaging attention from such stimuli. It was also expected that stronger involuntary processing of emotional expressions would be reflected in higher P1 amplitudes in high-anxiety participants compared with low-anxiety participants. This effect was anticipated for trials with threat-related expressions, but not for those involving neutral or positive expressions. Effects related to anxiety level were expected to be absent or substantially weaker for the N170 amplitude, given that no consistent anxiety-related modulation of the N170 has been reported to date. At the same time, the N170 amplitude was expected to vary depending on the emotional expression of the presented face. Any effect of emotional expression on the P1 amplitude was expected to be markedly weaker. Finally, neither the anxiety effect nor the emotional expression effect was expected to be observed in trials with inverted faces. The absence of such effects would be attributable to the strong disruption of face processing induced by face inversion, including impairments in face identification and in the recognition of emotional expressions. 

No specific hypotheses regarding anxiety-related variability in P1 and N170 latencies were formulated, as previous research has not demonstrated effects of anxiety level on the latencies of early ERP components. Analyses of P1 and N170 amplitudes and latencies were therefore conducted primarily to verify the effectiveness of the experimental manipulation (stimulus inversion).

## 2. Materials and Methods

### 2.1. Participants

A group of 51 students from Jagiellonian University, comprising 34 females and 17 males, participated as volunteers in this study. They were recruited through advertisements posted on campus. All participants were right-handed and had normal or corrected-to-normal vision. They were also screened to ensure that they were free from neurological or psychiatric disorders and were not regularly taking medication affecting the activity of the central nervous system. Participation was compensated with course credits. This study was approved by the Ethics Committee of the Institute of Psychology at Jagiellonian University. Prior to the experiment, all participants had given informed consent in accordance with the Declaration of Helsinki.

Data from three participants had to be excluded from the analysis due to technical problems during the EEG session, resulting in a final sample of 48 participants (31 females and 17 males; mean age = 21.29 years, SD = 1.71). 

Two groups were formed based on scores from the trait scale of the State-Trait Anxiety Inventory [48], using the Polish adaptation [49]. STAI scores in the full sample were normally distributed (skewness = −0.14; kurtosis = −0.41) and ranged from 23 to 59 (M = 42.06, SD = 8.36). The Low Anxiety (LA) group (n = 25; 18 females and 7 males) scored at or below the median (Md = 42), whereas the High Anxiety (HA) group (n = 23; 13 females and 10 males) scored above the median (M = 35.6, SD = 5.1, and M = 49.1, SD = 4.5 for the LA and HA groups, respectively). The initial analyses revealed no significant effects of gender; therefore, this factor was not included in subsequent analyses. The groups were comparable in mean age (LA: M = 20.8, SD = 1.3; HA: M = 21.6, SD = 1.9). A one-factor ANOVA confirmed a statistically significant difference in trait anxiety between the LA and HA groups (F(1,47) = 93.7, *p* < 0.0001). 

### 2.2. Stimuli and Procedure

Participants completed the STAI questionnaire at the beginning of the experimental session. During the EEG session, they were seated comfortably in a dimly lit, air-conditioned, and electrically shielded chamber. A computer screen was positioned approximately 70 cm in front of them.

Each trial began with the central presentation of a fixation cross for 500 ms, followed by the display of a face stimulus for 1000 ms. In half of the trials, an additional stimulus (a red asterisk symbol) was briefly presented for 50 ms in the centre of the screen during the face presentation. This served as the probe-target. The onset of the probe-target varied across trials (SOA = 400 ms, 550 ms, or 700 ms after the onset of the face stimulus). Participants were instructed to respond with their dominant hand by pressing a button whenever they detected the probe-target and to withhold responses when no such stimulus was presented. They were also instructed to maintain central fixation and to minimise eye blinks and excessive body movements during the EEG session.

Colour photographs of faces from 10 different individuals (5 men and 5 women), taken from the NimStim Face Stimulus Set [50] (http://www.macbrain.org/resources.htm, accessed on 10 April 2012), were used as stimuli. For each of the 10 individuals, one photograph was used for each of the tested emotional expressions. The facial expressions were fearful, angry, happy, or neutral, resulting in a total of 40 distinct face stimuli.

The task comprised 480 trials presented in a random order. To minimise fatigue, the experimental procedure was divided into three blocks of 160 trials each, counterbalanced with respect to trial type. Face stimuli were presented in the upright position in 240 trials, while in the remaining half, they were shown inverted (upside down). For upright faces, each emotional expression (fearful, angry, happy, and neutral) was presented in 60 trials per category. The same number of trials per category was used for inverted faces, with each expression also presented 60 times. Trials were also fully counterbalanced with respect to model identity and stimulus position. Each of the 10 images for a given model was presented 6 times in the upright position and 6 times in the inverted position.

The images presented on the screen measured 18 cm in height and 13 cm in width, corresponding to visual angles of 14.65° and 10.61°, respectively. The distance between the participant and the screen was approximately 70 cm. 

### 2.3. ERP Processing and Analyses

EEG was recorded using a BioSemi ActiveTwo system (BioSemi B.V., Amsterdam, The Netherlands) with Ag–AgCl electrodes from 64 monopolar locations arranged according to the extended 10–20 system. Two additional electrodes—the common mode sense (CMS) active electrode and the driven right leg (DRL) passive electrode—served as reference and ground, respectively (cf. www.biosemi.com/faq/cms&drl.htm, accessed on 6 May 2019). All cephalic electrodes were positioned on the scalp using an Electro-Cap. Two further electrodes were placed at the mastoids and were also referenced to the CMS active electrode. Horizontal and vertical EOGs were recorded using four electrodes positioned above and below the right eye and at the external canthi of both eyes.

EEG signals were acquired at a sampling rate of 512 Hz. Data were filtered offline with a 0.1–75 Hz (24 dB) bandpass filter and segmented into 600 ms epochs (150 ms prior to stimulus onset and 450 ms post-stimulus) using BrainVision Analyzer 2.0 (Brain Products GmbH, Gilching, Germany). Data were then corrected for eye-movement artefacts using Gratton–Cole’s regression method [51] and re-referenced to the linked mastoids. Separate averages were computed for all combinations of EMOTIONAL EXPRESSION (fearful vs. angry vs. happy vs. neutral) and FACE ORIENTATION (upright vs. inverted), resulting in eight averaged waveforms for each electrode and each participant.

Behavioural data were analysed using repeated-measures analysis of variance (ANOVA) with the within-subjects factors of EMOTIONAL EXPRESSION (fearful vs. angry vs. happy vs. neutral) and FACE ORIENTATION (upright vs. inverted), and the between-subjects factor of GROUP (LA vs. HA). Separate analyses were conducted for reaction times (RTs) and error rates (ERs) in trials containing the probe-target stimulus (red asterisk), as well as for false alarms (FA) in trials without target presentation. Initial analyses indicated that SOA did not modulate anxiety-related effects; therefore, this factor was not included in subsequent analyses.

Two separate sets of analyses were conducted for the EEG data. The first set examined peak latency and peak amplitude values for two canonical components, P1 and N170, recorded in response to face presentation. The P1 component was defined as the largest positive-going peak occurring 80–160 ms after face onset. Analyses of P1 latencies and amplitudes were performed on data from four occipital and occipito-temporal electrodes pooled together (O1, PO7, O2, and PO8). The N170 component was defined as the largest negative-going peak occurring 140–200 ms after face onset. Analyses of the N170 component were restricted to six occipito-temporal electrodes pooled together (PO7, P7, P9, PO8, P8, and P10). Data were analysed using repeated-measures ANOVA with the within-subjects factors of EMOTIONAL EXPRESSION (fearful vs. angry vs. happy vs. neutral), FACE ORIENTATION (upright vs. inverted), and electrode LOCATION (left hemisphere vs. right hemisphere), and the between-subjects factor of GROUP (LA vs. HA).

The average number of artefact-free trials was comparable across all experimental conditions. The number of trials obtained from individual participants included in the analysis of P1 and N170 ranged from 42 to 60 across experimental conditions. Across all eight experimental conditions, the average number of trials exceeded 58, with standard deviations below 5. No significant differences were observed as a function of emotional expression or stimulus orientation. Likewise, the two groups did not differ in the number of accepted trials per condition.

The second set of analyses examined differences in brain activity related to the processing of the probe-target stimuli (red asterisk). These analyses were based on mean voltage values within the 260–340 ms post-stimulus interval, corresponding to the P3 component, recorded from six central–parietal (CP1, CPz, CP2) and parietal (P1, Pz, P2) electrodes pooled together. Data were analysed using repeated-measures ANOVA with the within-subjects factors of EMOTIONAL EXPRESSION (fearful vs. angry vs. happy vs. neutral) and FACE ORIENTATION (upright vs. inverted), and the between-subjects factor of GROUP (LA vs. HA). Initial analyses indicated that SOA did not modulate anxiety-related effects; therefore, this factor was not included in subsequent analyses.

For all statistically significant results (*p* < 0.05) and nonsignificant trends (*p* < 0.10), effect sizes were reported as Cohen’s *f*. For all analyses, Greenhouse–Geisser corrections were applied to the degrees of freedom when appropriate, and only corrected probability values are reported. The Bonferroni method was used for post hoc comparisons, with a significance level of 0.05.

## 3. Results

**Figure 1:**
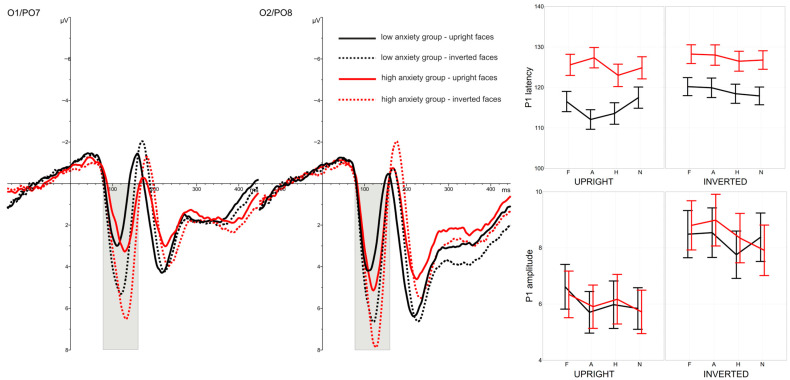
Grand-average ERPs recorded at left (O1/PO7) and right (O2/PO8) parieto-occipital electrodes in response to upright (solid lines) and inverted faces (dashed lines). Black lines represent responses from the low-anxiety group, and red lines represent responses from the high-anxiety group. The time window corresponding to the P1 component (80–160 ms post-stimulus) is highlighted at the relevant electrodes. Right panel: mean P1 latencies (top) and amplitudes (bottom), with SEM, plotted as a function of emotional expression (X-axis: F—fearful, A—angry, H—happy, N—neutral) and face orientation (upright vs. inverted). Black lines show values for the low-anxiety group and red lines for the high-anxiety group.

**Figure 2:**
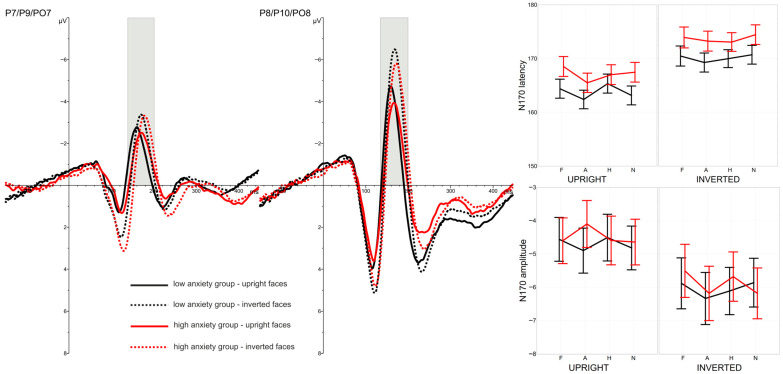
Grand-average ERPs recorded at left (P7/P9/PO7) and right (P8/P10/PO8) parieto-occipital electrodes in response to upright (solid lines) and inverted faces (dashed lines). Black lines represent responses from the low-anxiety group, and red lines represent responses from the high-anxiety group. The time window corresponding to the N170 component (140–200 ms post-stimulus) is highlighted at the relevant electrodes. Right panel: mean N170 latencies (top) and amplitudes (bottom), with SEM, plotted as a function of emotional expression (X-axis: F—fearful, A—angry, H—happy, N—neutral) and face orientation (upright vs. inverted). Black lines indicate values for the low-anxiety group and red lines for the high-anxiety group.

**Figure 3:**
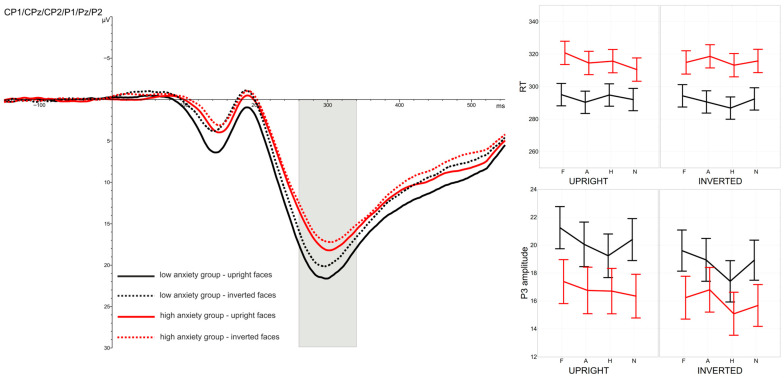
Grand-average ERPs recorded at central-parietal (CP1/CPz/CP2) and parietal (P1/Pz/P2) electrodes were pooled together in response to probe-targets presented following upright (solid lines) and inverted faces (dashed lines). Black lines indicate responses from the low-anxiety group, and red lines indicate responses from the high-anxiety group. The time window corresponding to the P3 component (260–340 ms post-stimulus) is highlighted at the relevant electrodes. Right panel: mean reaction times to probe-targets (top) and mean P3 amplitudes (bottom), with SEM, plotted as a function of emotional expression (X-axis: F—fearful, A—angry, H—happy, N—neutral) and face orientation (upright vs. inverted). Black lines represent values for the low-anxiety group and red lines for the high-anxiety group.

A Face Inversion Effect was evident at the early stages of processing for all emotional expressions tested and for both groups differing in anxiety level. Longer latencies and higher amplitudes of the P1 and N170 components were recorded in response to inverted faces compared with upright faces, as shown in Figure 1 and Figure 2. Face orientation also influenced brain activity elicited by the probe-targets: higher P3 amplitudes were observed in trials with upright faces. However, motor response speed to the probe-targets was not affected by face orientation (see Figure 3).

An effect of emotional expression was found at the initial stages of processing, as reflected in P1 latency. This effect occurred primarily when faces were presented upright and was observed only in the low-anxiety group. In contrast, emotional expressions influenced P1 amplitudes regardless of stimulus orientation, and this effect was present in both groups differing in STAI scores (see Figure 1). Additionally, anxiety level significantly affected P1 latencies across all conditions: longer latencies were recorded in the high-anxiety group, irrespective of face orientation or emotional expression.

N170 latency also varied depending on the emotional expression shown in the presented faces, but this effect was restricted to upright faces and occurred in both anxiety groups. In contrast, no emotional expression effect was observed for N170 amplitude. Furthermore, anxiety level had no influence on N170 latencies or amplitudes (see Figure 2).

Finally, an effect of emotional expression was also observed in P3 amplitude, and this effect was comparable across the two anxiety groups. 

All findings were confirmed by statistical analyses presented in the following subsections. Means and standard deviations for reaction times (RTs) and error rates are shown in Table 1. Means and standard deviations for amplitudes and latencies of the ERP components recorded in response to face presentation (P1 and N170), as well as mean P3 amplitudes elicited by the probe-targets, are shown in Table 2.

### 3.1. Behavioural Results

Correct responses to the probe-target were equally fast regardless of face orientation (304.25 ms vs. 303.36 ms for upright and inverted faces, respectively), as confirmed by a non-significant main effect of FACE ORIENTATION, *F*(1,46) = 0.34, *p* > 0.1, η2*p* = 0.01. At the same time, a non-significant trend for EMOTIONAL EXPRESSION was observed, *F*(3,138) = 2.39, *p* = 0.084, η2*p* = 0.05, Cohen’s *f* = 0.17, GG-ε = 0.819, together with a significant FACE ORIENTATION × EMOTIONAL EXPRESSION interaction, *F*(3,138) = 2.76, *p* = 0.047, η2*p* = 0.06, Cohen’s *f* = 0.19, GG-ε = 0.967.

A significant main effect of GROUP was also found, *F*(1,46) = 6.24, *p* = 0.016, η2*p* = 0.12, Cohen’s *f* = 0.33: participants with low anxiety responded faster on average (292.07 ms) than those with high trait anxiety (315.52 ms). This difference was stable and did not interact with any other factors. None of the interactions involving anxiety level reached significance: EMOTIONAL EXPRESSION × GROUP, *F*(3,138) = 0.94, *p* > 0.1, η2*p* = 0.02, GG-ε = 0.819; FACE ORIENTATION × GROUP, *F*(1,46) = 0.57, *p* > 0.1, η2*p* = 0.01; EMOTIONAL EXPRESSION × FACE ORIENTATION × GROUP, *F*(3,138) = 1.08, *p* > 0.1, η2*p* = 0.02, GG-ε = 0.967.

These results suggest that emotional expression affected response speed differently for upright and inverted faces. To verify this, we examined RTs separately for each orientation. When faces were presented upright, the fastest responses were observed for neutral expressions (301.27 ms) and the slowest for fearful expressions (307.95 ms). Responses to angry and happy faces were intermediate (302.49 ms and 305.28 ms, respectively). These differences yielded a significant effect of EMOTIONAL EXPRESSION, *F*(3,138) = 2.80, *p* = 0.043, η2*p* = 0.06, Cohen’s *f* = 0.19, GG-ε = 0.913.

In contrast, a non-significant trend for EMOTIONAL EXPRESSION was found in the inverted-face condition, *F*(3,138) = 2.29, *p* = 0.081, η2*p* = 0.05, Cohen’s *f* = 0.16, GG-ε = 0.929. Slightly faster responses were observed for happy faces (300.02 ms) compared to the other expressions (fearful: 304.63 ms; angry: 304.63 ms; neutral: 304.14 ms).

The overall number of errors was very low (below 2%). For omissions (missed probe-targets), incorrect responses occurred equally often regardless of EMOTIONAL EXPRESSION, *F*(3,138) = 1.71, *p* > 0.1, η2*p* = 0.04, GG-ε = 0.813, or FACE ORIENTATION, *F*(1,46) = 0.15, *p* > 0.1, η2*p* < 0.01. Their interaction was also non-significant, *F*(3,138) = 0.09, *p* > 0.1, η2*p* < 0.01, GG-ε = 0.829. Anxiety level did not influence the number of omissions, main effect of GROUP, *F*(1,46) = 0.09, *p* > 0.1, η2*p* < 0.01, nor did the interactions FACE ORIENTATION × GROUP, *F*(1,46) = 0.15, *p* > 0.1, η2*p* < 0.01 or FACE ORIENTATION × EMOTIONAL EXPRESSION × GROUP, *F*(3,138) = 1.17, *p* > 0.1, η2*p* = 0.02, GG-ε = 0.829.

The only significant result in the omission analysis concerned the EMOTIONAL EXPRESSION × GROUP interaction, *F*(3,138) = 2.98, *p* = 0.044, η2*p* = 0.06, Cohen’s *f* = 0.20, GG-ε = 0.813. Participants with low anxiety made the most errors when viewing angry faces (M = 1.67%) and the fewest for neutral expressions (M = 0.87%). In contrast, high-anxiety participants made the most errors for happy faces (M = 1.52%) and the fewest for angry faces (M = 0.65%). 

The analysis of false alarms revealed only one significant finding: participants made more false alarms when faces were presented upright (M = 0.91%) than inverted (M = 0.51%), producing a main effect of FACE ORIENTATION, *F*(1,46) = 8.51, *p* = 0.005, η2*p* = 0.16, Cohen’s *f* = 0.39. False alarms were not influenced by EMOTIONAL EXPRESSION, *F*(3,138) = 0.94, *p* > 0.1, η2*p* = 0.02, GG-ε = 0.949 or by GROUP, *F*(1,46) = 0.88, *p* > 0.1, η2*p* = 0.02. No other interactions reached significance (all *F* < 1.62, all *p* > 0.1).

### 3.2. Electrophysiological Results

#### 3.2.1. Latency of P1 Component

The time course of early responses to face stimuli differed depending on face orientation. The latency of the P1 component was shorter for upright faces (M = 120.06 ms) compared with inverted ones (M = 123.26 ms), yielding a significant main effect of FACE ORIENTATION, *F*(1,46) = 14.50, *p* < 0.001, η2*p* = 0.24, Cohen’s *f* = 0.53. This effect was similar in both anxiety groups, as indicated by the non-significant FACE ORIENTATION × GROUP interaction, *F*(1,46) = 1.46, *p* > 0.1, η2*p* = 0.03.

A significant main effect of EMOTIONAL EXPRESSION was also observed, *F*(3,138) = 3.08, *p* = 0.030, η2*p* = 0.06, Cohen’s *f* = 0.21, GG-ε = 0.964. This effect did not differ across face orientations, as shown by the non-significant EMOTIONAL EXPRESSION × FACE ORIENTATION interaction, *F*(3,138) = 1.67, *p* > 0.1, η2*p* = 0.03, GG-ε = 0.859. At the same time, emotional expression tended to affect the two anxiety groups differently, although this trend did not reach significance, EMOTIONAL EXPRESSION × GROUP, *F*(3,138) = 2.32, *p* = 0.078, η2*p* = 0.05, Cohen’s *f* = 0.17, GG-ε = 0.964. Importantly, this relationship varied depending on face orientation, resulting in a significant three-way EMOTIONAL EXPRESSION × FACE ORIENTATION × GROUP interaction, *F*(3,138) = 2.74, *p* = 0.046, η2*p* = 0.06, Cohen’s *f* = 0.19, GG-ε = 0.859.

A robust main effect of GROUP was also found, *F*(1,46) = 8.75, *p* = 0.005, η2*p* = 0.16, Cohen’s *f* = 0.40. Participants with low anxiety showed shorter P1 latencies overall (M = 117.03 ms) than those with high anxiety (M = 126.29 ms).

Taken together, these results indicate that the neural mechanisms underlying P1 generation may be differentially modulated by emotional expression and face orientation depending on anxiety level. To clarify this pattern, separate two-factor ANOVAs (EMOTIONAL EXPRESSION × FACE ORIENTATION) were conducted for each group.

The main effect of FACE ORIENTATION remained significant in both groups, low anxiety: *F*(1,24) = 10.69, *p* = 0.003, η2*p* = 0.31, Cohen’s *f* = 0.61; high anxiety: *F*(1,22) = 4.31, *p* = 0.050, η2*p* = 0.16, Cohen’s *f* = 0.37. However, the main effect of EMOTIONAL EXPRESSION did not reach significance in either group, low anxiety: *F*(3,72) = 2.71, *p* = 0.051, η2*p* = 0.10, Cohen’s *f* = 0.26, GG-ε = 0.928; high anxiety: *F*(3,66) = 2.68, *p* = 0.054, η2*p* = 0.11, Cohen’s *f* = 0.27, GG-ε = 0.877. Only the low-anxiety group showed a significant EMOTIONAL EXPRESSION × FACE ORIENTATION interaction, *F*(3,72) = 4.28, *p* = 0.008, η2*p* = 0.15, Cohen’s *f* = 0.36, GG-ε = 0.901, whereas this interaction was non-significant in the high-anxiety group, *F*(3,66) = 0.52, *p* > 0.1, η2*p* = 0.02, GG-ε = 0.639.

Further analyses revealed that in the low-anxiety group, P1 latency was influenced by EMOTIONAL EXPRESSION only for upright faces, *F*(3,72) = 4.00, *p* = 0.011, η2*p* = 0.14, Cohen’s *f* = 0.34, GG-ε = 0.929, not for inverted faces, *F*(3,72) = 2.14, *p* > 0.1, η2*p* = 0.08, GG-ε = 0.904. In contrast, emotional expression did not significantly influence P1 latencies in the high-anxiety group under either orientation, upright: *F*(3,66) = 1.92, *p* > 0.1, η2*p* = 0.08, GG-ε = 0.770; inverted: *F*(3,66) = 0.84, *p* > 0.1, η2*p* = 0.04, GG-ε = 0.815.

P1 latencies were comparable across hemispheres, with no significant main effect of LOCATION, *F*(1,46) = 1.30, *p* > 0.1, η2*p* = 0.03. Most interactions involving LOCATION were also non-significant (all *F* < 2.30, all *p* > 0.1). The sole exception was a significant LOCATION × EMOTIONAL EXPRESSION × GROUP interaction, *F*(3,138) = 3.81, *p* = 0.022, η2*p* = 0.08, Cohen’s *f* = 0.24, GG-ε = 0.738.

#### 3.2.2. Amplitude of P1 Component

The mode of face presentation had a highly significant effect on the amplitude of the P1 component. A robust main effect of FACE ORIENTATION was observed, *F*(1,46) = 134.07, *p* < 0.0001, η2*p* = 0.74, Cohen’s *f* = 1.66, with inverted faces eliciting higher amplitudes (M = 8.40 µV) than upright faces (M = 6.03 µV). This difference was not modulated by anxiety level, as indicated by the non-significant FACE ORIENTATION × GROUP interaction, *F*(1,46) = 0.29, *p* > 0.1, η2*p* = 0.01.

A significant main effect of EMOTIONAL EXPRESSION was also found, *F*(3,138) = 4.76, *p* = 0.003, η2*p* = 0.09, Cohen’s *f* = 0.28, GG-ε = 0.942, and this effect did not differ between anxiety groups, EMOTIONAL EXPRESSION × GROUP: *F*(3,138) = 1.66, *p* > 0.1, η2*p* = 0.03, GG-ε = 0.942. However, the influence of emotional expression varied depending on face orientation, resulting in a significant FACE ORIENTATION × EMOTIONAL EXPRESSION interaction, *F*(3,138) = 2.84, *p* = 0.040, η2*p* = 0.06, Cohen’s *f* = 0.20, GG-ε = 0.908. This relationship was not affected by anxiety level, as shown by the non-significant FACE ORIENTATION × EMOTIONAL EXPRESSION × GROUP interaction, *F*(3,138) = 0.68, *p* > 0.1, η2*p* = 0.01, GG-ε = 0.908. The main effect of GROUP was also non-significant, *F*(1,46) = 0.01, *p* > 0.1, η2*p* < 0.01, confirming that anxiety did not modulate P1 amplitude.

These findings indicate that emotional-expression–related variation in P1 amplitude differs depending on whether faces are presented upright or inverted. Therefore, two separate analyses were conducted for each orientation condition.

The main effect of EMOTIONAL EXPRESSION remained significant for both upright and inverted faces, upright: *F*(3,138) = 3.85, *p* = 0.011, η2*p* = 0.08, Cohen’s *f* = 0.24, GG-ε = 0.877; inverted: *F*(3,138) = 3.70, *p* = 0.013, η2*p* = 0.08, Cohen’s *f* = 0.24, GG-ε = 0.922. However, post hoc comparisons revealed distinct patterns across the two orientations. For upright faces, P1 amplitude was significantly larger in response to fearful expressions than to angry or neutral expressions (*p* = 0.009 and *p* = 0.002, respectively). For inverted faces, P1 amplitude was again greater for fearful expressions compared with happy or neutral expressions (*p* = 0.036 and *p* = 0.025, respectively), but responses to angry faces were also significantly larger than those elicited by happy or neutral faces (*p* = 0.011 and *p* = 0.032, respectively). 

Finally, P1 amplitude was larger over the right hemisphere (M = 8.17 µV) compared to the left (M = 6.27 µV), resulting in a significant main effect of LOCATION, *F*(1,46) = 17.94, *p* < 0.001, η2*p* = 0.28, Cohen’s *f* = 0.59. This hemispheric difference was comparable across anxiety groups, LOCATION × GROUP: *F*(1,46) = 0.15, *p* > 0.1, η2*p* < 0.01. All other interactions involving LOCATION were non-significant (all *F* < 2.15, all *p* > 0.1).

#### 3.2.3. Latency of N170 Component

A highly significant main effect of FACE ORIENTATION was observed for N170 latency, *F*(1,46) = 82.94, *p* < 0.0001, η2*p* = 0.64, Cohen’s *f* = 1.31. N170 latencies were shorter for upright faces (M = 165.49 ms) than for inverted faces (M = 171.91 ms). This effect was comparable across anxiety groups, as indicated by the non-significant FACE ORIENTATION × GROUP interaction, *F*(1,46) = 0.03, *p* > 0.1, η2*p* < 0.01.

A significant main effect of EMOTIONAL EXPRESSION was also found, *F*(3,138) = 3.95, *p* = 0.010, η2*p* = 0.08, Cohen’s *f* = 0.25, GG-ε = 0.823, and this effect did not differ between anxiety groups, EMOTIONAL EXPRESSION × GROUP: *F*(3,138) = 0.94, *p* > 0.1, η2*p* = 0.02, GG-ε = 0.823. The effect of emotional expression varied only marginally as a function of face orientation, yielding a non-significant trend for the FACE ORIENTATION × EMOTIONAL EXPRESSION interaction, *F*(3,138) = 2.29, *p* = 0.081, η2*p* = 0.05, Cohen’s *f* = 0.16, GG-ε = 0.851.

To further explore this interaction, separate analyses were conducted for upright and inverted faces. Significant differences among emotional expressions were found for upright faces, *F*(3,138) = 4.36, *p* = 0.009, η2*p* = 0.09, Cohen’s *f* = 0.27, GG-ε = 0.850, whereas no significant differences were observed for inverted faces, *F*(3,138) = 1.68, *p* > 0.1, η2*p* = 0.03, GG-ε = 0.915. The three-way interaction FACE ORIENTATION × EMOTIONAL EXPRESSION × GROUP was non-significant, *F*(3,138) = 0.61, *p* > 0.1, η2*p* = 0.01, GG-ε = 0.851, confirming that anxiety level did not modulate these effects. The main effect of GROUP was also non-significant, *F*(1,46) = 2.27, *p* > 0.1, η2*p* = 0.05. 

Finally, analyses involving the LOCATION factor revealed no significant hemispheric differences in N170 latency, nor any significant interactions with other variables (all *F* < 1.58, all *p* > 0.1).

#### 3.2.4. Amplitude of N170 Component

Higher N170 amplitudes were obtained in response to inverted faces (M = −5.97 µV) compared with upright faces (M = −4.59 µV), yielding a highly significant main effect of FACE ORIENTATION, *F*(1,46) = 31.57, *p* < 0.0001, η2*p* = 0.41, Cohen’s *f* = 0.80. N170 amplitudes were also substantially larger over the right hemisphere (M = −6.70 µV) than over the left (M = −3.86 µV), as indicated by a significant main effect of LOCATION, *F*(1,46) = 26.66, *p* < 0.0001, η2*p* = 0.37, Cohen’s *f* = 0.73. Moreover, the face inversion effect was more pronounced over the right hemisphere, resulting in a significant FACE ORIENTATION × LOCATION interaction, *F*(1,46) = 14.57, *p* < 0.001, η2*p* = 0.24, Cohen’s *f* = 0.53. 

No differential effect of emotional expression on N170 amplitude was observed: the main effect of EMOTIONAL EXPRESSION was non-significant, *F*(3,138) = 1.45, *p* > 0.1, η2*p* = 0.03, GG-ε = 0.941. Likewise, anxiety level had no influence on N170 amplitude, main effect of GROUP: *F*(1,46) = 0.04, *p* > 0.1, η2*p* < 0.01, and all interaction effects involving these factors were non-significant (all *F* < 2.00, all *p* > 0.1).

#### 3.2.5. Mean Amplitude of P3 Component (260–340 ms After Probe-Target Presentation)

Higher P3 amplitudes were recorded for participants with low STAI scores (M = 19.48 µV) compared with those in the high-anxiety group (M = 16.37 µV); however, this difference did not reach statistical significance, main effect of GROUP: *F*(1,46) = 2.18, *p* > 0.1, η2*p* = 0.04.

A significant main effect of FACE ORIENTATION was observed, *F*(1,46) = 30.12, *p* < 0.0001, η2*p* = 0.40, Cohen’s *f* = 0.78. P3 amplitudes elicited by probe-targets were higher following upright faces (M = 18.52 µV) than inverted faces (M = 17.33 µV). A significant main effect of EMOTIONAL EXPRESSION was also found, *F*(3,138) = 7.10, *p* < 0.001, η2*p* = 0.13, Cohen’s *f* = 0.36, GG-ε = 0.860. The largest amplitudes occurred when probe-targets were preceded by fearful faces (M = 18.62 µV), followed by angry (M = 18.14 µV) and neutral expressions (M = 17.84 µV), with the lowest amplitudes observed after happy faces (M = 17.11 µV). 

The effect of facial orientation was comparable across all emotional expressions, as confirmed by the non-significant FACE ORIENTATION × EMOTIONAL EXPRESSION interaction, *F*(3,138) = 1.50, *p* > 0.1, η2*p* = 0.03, GG-ε = 0.956. All remaining interaction effects were likewise non-significant (all *F* < 2.4, all *p* > 0.1).

## 4. Discussion

The aim of the present study was to examine anxiety-related differences in brain activity elicited by task-irrelevant faces displaying various emotional expressions presented in upright or inverted orientation. Additionally, ERPs were recorded in response to simple neutral visual stimuli (probe-targets) that were presented simultaneously with, but unpredictably relative to, the facial images.

In line with a substantial body of previous research, ERPs elicited by upright faces differed markedly from those evoked by inverted faces at parieto-occipital sites. Inversion produced longer latencies and larger amplitudes of the early P1 (80–160 ms post-stimulus) and N170 (140–200 ms post-stimulus) components. This effect was expected and confirmed the effectiveness of the experimental manipulation. Participants processed facial images differently depending on their orientation. It is widely established that inverted faces are recognised less efficiently due to impaired structural encoding, which is indexed by the N170 component [10,11,12]. The present results replicate this pattern and further support evidence that face inversion influences not only later stages of processing but also earlier perceptual stages, as reflected in the P1 component [25,26]. Presenting a face in an inverted position markedly alters its processing, beginning as early as ~80 ms post-stimulus, and this effect persisted for roughly the next 100 ms. The current findings therefore suggest that inversion-related disruptions are not restricted to later, more elaborate structural analyses of faces, but also manifest during earlier stages associated with initial face detection.

The early stage of face processing, indexed by P1 amplitude, was also significantly modulated by emotional expression in both anxiety groups. At the subsequent processing stage reflected in the N170, the influence of emotional expression was reduced. Differences in N170 latency across emotional categories remained detectable, but only for upright faces and independently of anxiety level. Emotional expression did not modulate the N170 amplitude. 

The obtained results are only partially consistent with the hypotheses formulated prior to the study. It was expected that effects related to emotional expression would be most pronounced in the N170 component and weaker or absent in the P1. Contrary to this expectation, the statistical analyses revealed that emotional expression modulated neural activity more strongly at an earlier stage of processing, as reflected by differences in P1 amplitude. A markedly weaker effect was observed at a later stage, limited to upright-oriented faces, as reflected in N170 latency. These findings nevertheless provide partial support for the hypothesis concerning the role of face inversion in modulating emotion-related effects. Specifically, it was predicted that inversion would disrupt face processing to such an extent that effects of emotional expression would not be observed for inverted faces, a prediction that was only partially supported by the present results.

It should be noted, however, that participants were not required to explicitly differentiate the emotional expressions in the presented photographs. Consequently, the observed variations in neural activity reflect involuntary processing. Under task conditions that required participants to focus attention on a specific region of the visual field and to monitor intensively the stimuli appearing within it, we nevertheless observed differential early brain responses to task-irrelevant faces displaying distinct emotional expressions. This early differentiation emerged in the time window of the P1 component and is consistent with previous reports demonstrating emotional modulation of early visual processing [40,52,53,54]. In contrast, emotional expression did not modulate the later stage of face analysis reflected in the N170. Similar null findings for N170 amplitude have frequently been reported in studies where emotional expression is not task-relevant (e.g., [22]; see also [24] for review).

Taken together, these results support the notion that emotional expression recognition involves at least two functionally distinct stages, which may differentially reflect involuntary and voluntary aspects of facial-emotion processing. The P1 component is commonly linked to low-level analysis of facial features [55,56]. Therefore, the early emotional expression effect likely reflects rapid detection of salient visual changes associated with specific emotions—for example, widened eyes in fearful faces. By contrast, the N170 is associated with more advanced configural processing, within-category differentiation [55], and the emergence of conscious perception of faces and their emotional content [57]. The present pattern thus reinforces the view that the processing of facial emotion is rapid, efficient, and prioritised—consistent with the high biological and social relevance of facial signals.

Moreover, involuntary detection of facial emotions does not preclude their influence on subsequent cognitive processes, such as the detection and response to a neutral visual stimulus. Behavioural and electrophysiological responses to task-relevant stimuli provided further support for these assumptions: reaction times to neutral probe-targets varied as a function of the emotional expression of the preceding face, and the P3 amplitude elicited by these stimuli also differed across emotional categories.

Anxiety-related differences in brain responses emerged at 80–160 ms post-stimulus, overlapping with the P1 component. Participants with higher anxiety exhibited longer P1 latencies, an effect that was independent of facial expression and orientation. In contrast, P1 amplitude did not differ as a function of anxiety level. The obtained results are therefore inconsistent with the hypothesis formulated prior to the study. A higher P1 amplitude was expected in the high-anxiety group. 

Previous reports have interpreted enhanced P1 responses in anxious individuals as reflecting increased sensitivity to threat signals [41] or heightened hypervigilance in social contexts [43]. More elaborate explanations for anxiety-related differences in P1 magnitude have been relatively rare. Some researchers have proposed that the effects observed in individuals with elevated anxiety resemble those identified in studies of spatial attention mechanisms, in which larger P1 amplitudes are recorded for attended compared to unattended stimuli. However, at the same time, no corresponding differences in behavioural measures (e.g., reaction times) were typically observed between groups differing in anxiety levels [40,41]. In explaining the source of P1 amplitude differences, some authors have hypothesised that the increased allocation of attentional resources to emotionally relevant stimuli may result from feedback signals sent from the amygdala to the visual cortex [40]. This explanation, however, was not directly tested in these studies.

It should be noted, however, that the absence of P1 amplitude differences has also been reported in several earlier studies, indicating that this effect is not consistently observed across research designs [44,45,54]. The findings of the present study likewise do not support the universality of such anxiety-related P1 amplitude enhancement. This raises the question of possible reasons for the discrepancies in the results reported by different researchers. Among the factors that may significantly contribute to discrepancies in findings are differences in participant selection, variations in methods used to record neural activity, and the wide diversity of experimental designs employed across studies. Some studies have relied on measures of generalised anxiety [40,44,45], whereas others have selected participants using scales targeting specific forms of anxiety (e.g., social anxiety) [41,42]. Although these constructs are closely related and scores on different anxiety measures are often strongly correlated, they may nonetheless manifest differently in laboratory settings, particularly with respect to physiological responses, task performance, and the processing of stimuli with high social relevance, such as faces.

Previous studies have also differed substantially in their EEG methodologies, including the use of different reference schemes (e.g., earlobe or mastoid references, average reference) and varying electrode selections for P1 amplitude analyses. Moreover, P1 effects have been quantified using either mean amplitudes or peak values across different time windows. Considerable variability also existed in the experimental tasks employed, ranging from emotional expression recognition in schematic faces [42], to dot-probe paradigms [41], repetition-detection tasks [40], and facial gender discrimination tasks [44], among others. Taken together, these considerations suggest that the likelihood of observing anxiety-related differences in P1 amplitude depends on multiple methodological and experimental factors that have not always been adequately controlled in some earlier studies [40,41,42]. More recent investigations examining neural responses across several tasks performed by the same participants either fail to demonstrate reliable P1 differences between individuals with varying anxiety levels [44,45] or may even suggest reduced specificity of early visual processing stages in individuals with elevated anxiety [47,54].

The absence of an effect of anxiety on P1 amplitude does not necessarily indicate that early-stage processing is comparable across individuals differing in anxiety. The present results suggest that anxiety-related variation may instead be reflected in the time course of face processing. Participants with higher STAI scores exhibited systematically longer P1 latencies, and this effect was evident for all emotional expressions and both stimulus orientations. This finding was not expected. No a priori hypotheses were formulated regarding the influence of anxiety level on the latencies of the early P1 and N170 components elicited by face presentation. Analyses of component latencies were therefore primarily intended to verify the effectiveness of the face orientation manipulation.

It is also worth emphasising that the vast majority of previous reports have focused on differences in P1 amplitude and did not examine, or at least did not report, latency measures [40,41,42,43,44,45,46,47,54]. Consequently, it remains unclear whether latency analyses were not performed or whether they failed to reveal systematic anxiety-related effects and were therefore omitted. For this reason, the present findings should be interpreted with caution, and independent replication in future studies is clearly warranted.

Anxiety-related differences in P1 latency constitute a novel finding of the present study. Accordingly, their interpretation remains speculative and should be treated with caution, and replication in future research is clearly required. One possible explanation for the prolonged P1 latencies observed in the high-anxiety group is a general slowing of information processing. This interpretation is consistent with the fact that the anxiety effect was observed irrespective of emotional expression and face orientation. Processing delay could ultimately contribute to the longer behavioural reaction times observed in response to probe-target stimuli. 

However, several findings from the present study are inconsistent with this explanation. No significant differences were observed in the latency of the N170 component, suggesting that if slower processing characterises individuals with higher levels of anxiety, it is confined to very early stages of perceptual analysis rather than reflecting a general slowing of information processing. Moreover, the initial rise in the P1 component appeared comparable across groups, which argues against a broad alteration in early visual processing dynamics. Finally, if elevated anxiety were associated with a generalised slowing of information processing leading to longer reaction times, similar effects should have been consistently reported in previous studies employing comparable paradigms. However, the existing literature does not provide clear evidence for a systematic presence of such anxiety-related differences [40,41,42,44,45,54].

An alternative explanation assumes no differences in the overall speed of information processing in individuals with elevated levels of anxiety. Instead, the observed differences in P1 latency may reflect a prolonged neuronal activity underlying the generation of this component, resulting in a later occurrence of the waveform peak. Subsequent stages of processing, indexed by N170 component, do not exhibit significant differences in temporal characteristics between groups with varying anxiety levels. Prolonged early neuronal activity, however, may be associated with stronger involuntary engagement in stimulus processing, leading to greater difficulty in disengaging from the stimulus and reduced efficiency in responding to subsequent events. Similar behavioural patterns have been reported in previous studies [33,34,35,36]. In the present study, highly anxious individuals responded significantly more slowly to unpredictable probe-targets, and this behavioural difference co-occurred with a tendency toward reduced P3 amplitudes in the high-anxiety group. Previous studies have indicated a possible reduction in P3 amplitude following exposure to a task-irrelevant preceding stimulus, which has been interpreted as reflecting increased engagement in processing that stimulus and a consequent limitation in processing the subsequent target stimulus [58]. This hypothesis may be partially supported by the results of the correlation analyses presented in the Supplementary Materials. 

A potential neural source of such prolonged activity may be increased reactivity of limbic structures, particularly the amygdala. This interpretation remains highly speculative and warrants further empirical testing.

The hypotheses formulated prior to the study also considered the possibility that anxiety level would differentially modulate P1 amplitude as a function of emotional expression. Specifically, it was expected that expressions associated with threat (fear and anger) would produce a stronger influence of high anxiety compared to positive or neutral expressions. Such a pattern would support the notion that individuals with elevated anxiety exhibit increased sensitivity to threat-related signals. The results of the present study do not support these hypotheses. No significant differences in P1 amplitude were observed between groups with differing anxiety levels, nor was there evidence that threat-related expressions produced a more pronounced effect in the high-anxiety group. Instead, elevated anxiety level was associated with prolonged P1 latencies, an effect that was observed irrespective of emotional expression or stimulus orientation. This pattern suggests that anxiety-related modulation occurred in response to all presented stimuli. 

The hypothesis that the effects of high anxiety would be primarily observed for threat-related expressions assumes that the early P1 effect may reflect a hypervigilant tendency characteristic of highly anxious individuals, particularly toward visually salient stimuli with evolutionary significance, such as threat-related cues. Moreover, this early ERP modulation may be linked to enhanced sensory encoding in visual cortical areas driven by rapid feedback projections from the amygdala, where initial evaluation of emotionally significant stimuli occurs [59]. Numerous neuroimaging studies support this interpretation, demonstrating stronger amygdala responses in highly trait-anxious individuals when processing task-irrelevant fearful stimuli compared to low-anxious individuals [60,61,62]. Elevated amygdala activity to fearful relative to happy faces can occur even under backward-masking conditions that prevent conscious recognition [63]. 

This hypothesis further implies that the effect should diminish for positive or neutral expressions. However, when the emotional content of upcoming stimuli is unpredictable, this modulation may not be stimulus-specific. Under such conditions, unsystematic exposure to threat-related expressions across trials could lead to sustained amygdala activation, thereby influencing early visual processing more generally. In other words, detection of an unpredictable and potentially threatening visual signal may trigger prolonged amygdala activation in high-anxious individuals, which subsequently modulates the processing of all visual stimuli that follow, regardless of their emotional content or orientation. This mechanism provides a plausible explanation for why the anxiety-related effects observed in the present study appeared consistently across all tested emotional expressions and were independent of face orientation. However, it should be noted that this relationship was not directly tested in the present study. Moreover, EEG does not allow for direct assessment of activity in subcortical structures such as the amygdala; therefore, this interpretation remains speculative. 

Taken together, the findings of the present study indicate that early phases of visual information processing are substantially altered in individuals with elevated anxiety levels. Most notably, the high-anxiety group demonstrated longer P1 latencies, suggesting a difference in early perceptual response to faces. It is plausible that the early anxiety-related modulation overlapping with the P1 component may be, at least partially, functionally linked to the later behavioural slowing observed in responses to probe-targets. This interpretation highlights the potential cascade of effects initiated at early perceptual stages, ultimately influencing the efficiency of subsequent stimulus processing.

Individuals in the high-STAI group exhibited significantly longer reaction times to neutral probe-targets presented simultaneously with face stimuli. This effect was independent of both the emotional expression and the orientation of the faces, suggesting that it may stem from stronger involuntary attentional engagement elicited by the facial stimuli in highly anxious individuals. As a consequence, these participants may have greater difficulty disengaging attention from task-irrelevant faces, which in turn interferes with the processing of subsequently presented probe-targets.

Alterations in early visual information processing (indexed by the P1 component) may be associated with downstream deficits in face processing, as reflected in longer reaction times. To some extent, the anxiety-related alterations in early processing observed in the present study may correspond to the emotion recognition deficits reported by other researchers. Although evidence from individual studies is mixed—with many reporting comparable performance between high- and low-anxiety participants—recent meta-analyses that integrate larger bodies of empirical work indicate that significant impairments in emotion recognition are indeed present among individuals with elevated anxiety levels or anxiety disorders [64,65,66].

### 4.1. Study Limitations and Future Directions

Despite its strengths, the present study has several limitations that should be taken into account when interpreting the findings. First, although the sample size is typical for ERP research, it may have been insufficient to detect subtle or higher-order interaction effects, particularly those involving individual differences in anxiety. Second, emotional expressions were task-irrelevant, which—while increasing ecological validity—may have reduced the sensitivity of some ERP components (notably N170) to emotional content, limiting conclusions about voluntary or attention-driven emotion processing. Third, only static facial photographs were used, despite evidence that dynamic expressions elicit more robust and naturalistic neural responses. Fourth, the division into high- and low-anxiety groups was based on a median split of STAI scores, which reduces variability, as well as overall statistical power of analyses, and may obscure continuous relationships between anxiety and neural indices. Fifth, the EEG system allowed precise measurement of scalp-level components but did not permit source localization, especially for subcortical brain structures; thus, interpretations regarding specific brain structures, such as the amygdala, remain indirect. Finally, the experimental task, which involved monitoring probe-targets while ignoring faces, represents a relatively controlled and artificial context; therefore, the generalizability of the results to everyday social perception or emotionally demanding environments is limited. 

Future studies should therefore consider larger samples and analytic approaches that retain anxiety as a continuous variable to improve sensitivity to individual differences. Incorporating dynamic facial stimuli or more complex, socially relevant tasks could provide a more ecologically valid assessment of emotional processing. Combining ERP with neuroimaging techniques capable of spatial localization (e.g., fMRI or EEG-fMRI integration) may help clarify the neural mechanisms underlying early anxiety-related effects, particularly the role of amygdala-visual interactions. Additionally, manipulations of attentional relevance—such as tasks requiring explicit emotion recognition—may help disentangle the contributions of automatic versus controlled processes across early and late stages of face perception. Together, such methodological refinements would allow a more comprehensive understanding of how anxiety shapes the temporal dynamics of emotional face processing.

### 4.2. Conclusions

The study revealed anxiety-related modulation of early emotional face processing, beginning at approximately 100 ms post-stimulus within the P1 time window. Specifically, individuals with higher anxiety exhibited delayed P1 latencies compared to low-anxiety participants, indicating distinct patterns of early face processing between the groups. This effect was independent of emotional expression and stimulus orientation. Moreover, alterations in early face processing were indirectly associated with responses to the subsequently presented neutral visual stimulus: individuals with higher anxiety demonstrated longer reaction times, suggesting greater difficulty disengaging attention from expressive faces.

## Figures and Tables

**Table 1:** Mean response times (RTs; with SD) to probe-targets and error rates (omissions and false alarms) for each experimental condition, presented separately for the low- and high-anxiety groups as defined by STAI scores.

	Upright Faces				Inverted Faces			
Group	Fearful Faces	Angry Faces	Happy Faces	Neutral Faces	Fearful Faces	Angry Faces	Happy Faces	Neutral Faces
	Reaction times (ms)							
Low anxiety	295.10 (29.92)	290.38 (31.42)	294.86 (33.57)	292.05 (23.55)	294.35 (25.42)	290.60 (24.29)	286.81 (29.66)	292.45 (27.68)
High anxiety	320.81 (37.31)	314.59 (38.44)	315.71 (40.58)	310.42 (32.50)	314.91 (38.44)	318.67 (40.08)	313.23 (41.05)	315.83 (42.18)
	Omissions (error-rate)							
Low anxiety	1.20 (3.58)	1.73 (3.06)	0.93 (2.81)	0.80 (2.93)	0.93 (2.81)	1.60 (3.21)	1.60 (3.06)	0.93 (2.81)
High anxiety	0.87 (2.88)	0.58 (1.64)	1.74 (3.31)	0.87 (1.50)	1.01 (1.86)	0.72 (1.73)	1.30 (2.79)	1.01 (1.86)
	False alarms (error-rate)							
Low anxiety	0.67 (2.15)	1.20 (2.33)	0.67 (1.67)	0.53 (1.25)	0.53 (1.57)	0.53 (1.57)	0.27 (0.92)	0.27 (0.92)
High anxiety	1.30 (2.41)	1.01 (2.12)	1.01 (2.34)	0.87 (1.80)	1.16 (1.91)	0.14 (0.69)	0.29 (0.96)	0.87 (1.50)

**Table 2:** Mean latencies and amplitudes (with SD) of the P1 and N170 components elicited by face presentations, and mean P3 amplitudes measured in response to probe-targets, for each condition and for both groups differing in STAI scores.

		Upright Faces				Inverted Faces			
Group		Fearful Faces	Angry Faces	Happy Faces	Neutral Faces	Fearful Faces	Angry Faces	Happy Faces	Neutral Faces
		Latency P1 (80–160 ms post-stimulus; ms)							
Low anxiety	Left	117.03 (16.20)	110.47 (12.40)	112.68 (13.40)	117.34 (17.86)	118.51 (13.64)	119.61 (14.26)	119.14 (15.13)	119.02 (13.80)
	Right	116.01 (16.93)	113.71 (15.51)	114.45 (15.75)	117.66 (16.97)	121.91 (16.22)	120.23 (16.25)	117.77 (15.19)	116.83 (14.40)
High anxiety	Left	129.50 (13.02)	129.37 (13.51)	124.40 (16.71)	124.74 (13.82)	131.50 (11.27)	131.41 (13.49)	127.93 (12.41)	128.44 (10.09)
	Right	121.69 (11.23)	125.34 (13.36)	121.60 (16.58)	124.96 (11.74)	125.00 (9.57)	124.62 (9.66)	125.04 (11.67)	125.13 (12.15)
		Amplitude P1 (80–160 ms post-stimulus; μV)							
Low anxiety	Left	5.37 (3.49)	4.61 (3.86)	4.89 (3.92)	5.04 (3.81)	7.36 (3.89)	7.49 (4.16)	6.76 (3.86)	7.46 (4.33)
	Right	7.85 (5.92)	6.79 (5.52)	7.06 (6.03)	6.63 (5.33)	9.62 (6.64)	9.59 (6.03)	8.74 (6.22)	9.30 (6.73)
High anxiety	Left	5.20 (3.53)	4.93 (3.01)	5.20 (3.82)	4.52 (3.18)	8.18 (3.99)	8.12 (4.51)	7.90 (3.97)	7.20 (3.78)
	Right	7.48 (3.83)	6.88 (3.26)	7.14 (3.90)	6.91 (3.27)	9.42 (3.34)	9.86 (3.83)	8.79 (3.49)	8.63 (3.23)
		Latency N170 (140–200 ms post-stimulus; ms)							
Low anxiety	Left	164.63 (11.78)	163.98 (9.51)	162.68 (8.95)	164.04 (10.09)	170.57 (9.57)	171.71 (8.97)	170.10 (10.00)	171.33 (10.18)
	Right	166.09 (12.21)	164.84 (12.47)	162.13 (11.86)	162.29 (11.57)	169.43 (10.59)	169.27 (10.42)	168.46 (9.80)	170.13 (10.03)
High anxiety	Left	168.53 (9.81)	168.99 (10.91)	166.55 (10.69)	167.88 (11.04)	174.17 (8.15)	175.04 (10.35)	175.30 (10.45)	176.35 (9.69)
	Right	165.51 (10.55)	168.11 (11.53)	164.46 (11.39)	167.06 (11.27)	172.02 (9.64)	172.84 (10.75)	171.22 (10.06)	172.58 (9.48)
		Amplitude N170 (140–200 ms post-stimulus; μV)							
Low anxiety	Left	−3.49 (3.01)	−3.27 (3.06)	−3.53 (3.08)	−3.66 (2.82)	−4.24 (3.26)	−4.15 (3.46)	−4.59 (3.23)	−4.04 (2.96)
	Right	−5.53 (4.58)	−5.85 (4.43)	−6.27 (4.16)	−5.98 (4.18)	−7.99 (4.87)	−7.61 (5.28)	−8.08 (5.19)	−7.68 (4.58)
High anxiety	Left	−3.59 (3.09)	−3.30 (3.22)	−3.08 (3.08)	−3.78 (2.83)	−4.07 (3.66)	−4.02 (3.56)	−4.36 (3.88)	−4.58 (3.83)
	Right	−5.61 (4.78)	−5.90 (4.44)	−5.12 (5.00)	−5.51 (4.74)	−7.29 (4.50)	−6.99 (4.86)	−8.00 (5.31)	−7.79 (5.39)
		Mean Amplitude P3 (260–340 ms post-stimulus; μV)							
Low anxiety		21.25 (8.35)	20.06 (8.29)	19.24 (7.79)	20.40 (7.76)	19.61 (7.69)	18.94 (7.79)	17.41 (7.59)	18.92 (7.54)
High anxiety		17.39 (6.58)	16.75 (7.64)	16.71 (7.81)	16.35 (7.25)	16.23 (7.00)	16.80 (7.57)	15.08 (7.19)	15.67 (6.78)

## Data Availability

The raw data supporting the conclusions of this article will be made available by the author on request.

## Supplementary Materials

The following supporting information can be downloaded at: https://www.mdpi.com/article/10.3390/brainsci16010026/s1, Table S1. Pearson’s correlation coefficients (one-tailed) between STAI scores, P1 latencies and mean P3 amplitudes for each experimental condition across all participants (n = 48); Table S2. Pearson’s correlation coefficients (one-tailed) between STAI scores, P1 latencies and Reaction Times (RT) for each experimental condition across all participants (n = 48); Table S3. Pearson’s correlation coefficients (one-tailed) between STAI scores, mean P3 amplitudes and Reaction Times (RT) for each experimental condition across all participants (n = 48).

## Abbreviations

The following abbreviations are used in this manuscript:

ERP	Event-Related Potentials
EEG	Electroencephalography
STAI	State-Trait Anxiety Inventory
fMRI	Functional Magnetic Resonance Imaging
CMS	Common Mode Sense
LPP	Late Positive Potential
RT	Reaction Time
