# Supplementary material for: Anxiety-Related Modulation of Early Neural Responses to Task-Irrelevant Emotional Faces
Source: Brain Sci. 2025 Dec 25;16(1):26. doi: 10.3390/brainsci16010026 (PMC12839380; doi:10.3390/brainsci16010026)
Supplement: Supplementary file 1 [file brainsci-16-00026-s001.zip › brainsci-4022354-supplementary.pdf]

Table S1. Pearson's correlation coefficients (one-tailed) between STAI scores, P1 latencies and mean P3 amplitudes for each experimental condition across all participants (N = 48).

	STAI	P1 latency<br>Fearful upright	P1 latency<br>Angry upright	P1 latency<br>Happy upright	P1 latency<br>Neutral upright	P1 latency<br>Fearful inverted	P1 latency<br>Angry inverted	P1 latency<br>Happy inverted	P1 latency<br>Neutral inverted
STAI		**,271***<br>**p=,031**	**,396****<br>**p=,003**	**,288***<br>**p=,023**	,183<br>n.s.	,234<br>p=,055	**,240***<br>**p=,050**	,219<br>p=,067	**,263***<br>**p=,035**
P3 mean amplitude<br>Fearful – upright	-,183<br>n.s.	-,167<br>n.s.	-,233<br>p=,055	**-,310***<br>p=**,016**	**-,262***<br>p=**,036**	-,222<br>p=,065	**-,306***<br>p=**,017**	**-,244***<br>p=**,047**	**-,243***<br>p=**,048**
P3 mean amplitude<br>Angry – upright	-,155<br>n.s.	-,231<br>p=,057	-,239<br>p=,051	**-,305***<br>p=**,018**	**-,308***<br>p=**,017**	**-,264***<br>p=**,035**	**-,352****<br>p=**,007**	**-,305***<br>p=**,018**	**-,276***<br>p=**,029**
P3 mean amplitude<br>Happy – upright	-,105<br>n.s.	-,202<br>p=,085	-,154<br>n.s.	**-,276***<br>p=**,029**	-,208<br>p=,078	-,238<br>p=,052	**-,268***<br>p=**,033**	**-,284***<br>p=**,025**	-,227<br>p=,060
P3 mean amplitude<br>Neutral – upright	-,161<br>n.s.	-,146<br>n.s.	-,174<br>n.s.	**-,269***<br>p=**,032**	-,207<br>p=,080	-,198<br>p=,089	**-,247***<br>p=**,045**	-,230<br>p=,058	-,205<br>p=,081
P3 mean amplitude<br>Fearful – inverted	-,170<br>n.s.	-,209<br>p=,077	-,200<br>p=,087	**-,283***<br>p=**,026**	**-,248***<br>p=**,045**	-,227<br>p=,061	**-,297***<br>p=**,020**	-,220<br>p=,067	-,225<br>p=,062
P3 mean amplitude<br>Angry – inverted	-,108<br>n.s.	**-,265***<br>p=**,034**	-,175<br>n.s.	**-,331***<br>p=**,011**	**-,282***<br>p=**,026**	**-,275***<br>p=**,029**	**-,335****<br>p=,010	**-,307***<br>p=**,017**	**-,242***<br>p=**,048**
P3 mean amplitude<br>Happy – inverted	-,105<br>n.s.	**-,271***<br>p=**,031**	-,193<br>p=,094	**-,375****<br>p=**,004**	**-,328***<br>p=**,011**	**-,317***<br>p=**,014**	**-,321***<br>p=**,013**	**-,318***<br>p=**,014**	**-,278***<br>p=**,028**
P3 mean amplitude<br>Neutral - inverted	-,186<br>n.s.	-,164<br>n.s.	-,144<br>n.s.	**-,266***<br>p=**,034**	-,205<br>p=,081	-,196<br>p=,091	**-,250***<br>p=**,044**	-,193<br>p=,094	-,200<br>p=,086

Table S2. Pearson's correlation coefficients (one-tailed) between STAI scores, P1 latencies and Reaction Times (RT) for each experimental condition across all participants (N = 48).

	STAI	P1 latency<br>Fearful upright	P1 latency<br>Angry upright	P1 latency<br>Happy upright	P1 latency<br>Neutral upright	P1 latency<br>Fearful inverted	P1 latency<br>Angry inverted	P1 latency<br>Happy inverted	P1 latency<br>Neutral inverted
STAI		**,271***<br>**p=,031**	**,396****<br>**p=,003**	**,288***<br>**p=,023**	,183<br>n.s.	,234<br>p=,055	**,240***<br>**p=,050**	,219<br>p=,067	**,263***<br>**p=,035**
RT - Fearful - upright	**,288***<br>**p=,023**	,169<br>n.s.	**,318***<br>**p=,014**	,148<br>n.s.	,186<br>n.s.	**,256***<br>**p=,039**	,215<br>p=,071	,183<br>n.s.	**,243***<br>**p=,048**
RT - Angry - upright	,230<br>p=,058	,159<br>n.s.	**,249***<br>**p=,044**	,174<br>n.s.	,142<br>n.s.	**,241***<br>**p=,050**	,216<br>p=,071	,212<br>p=,074	,239<br>p=,051
RT - Happy - upright	,221<br>p=,066	,132<br>n.s.	**,246***<br>**p=,046**	,132<br>n.s.	,119<br>n.s.	,224<br>p=,063	,204<br>p=,082	,184<br>n.s.	,227<br>p=,061
RT - Neutral - upright	**,246***<br>**p=,046**	,194<br>p=,094	**,289***<br>**p=,023**	,207<br>p=,079	,202<br>p=,084	**,268***<br>**p=,033**	,203<br>p=,083	,201<br>p=,086	**,242***<br>**p=,048**
RT - Fearful - inverted	,221<br>p=,066	,101<br>n.s.	,229<br>p=,059	,080<br>n.s.	,142<br>n.s.	,215<br>p=,071	,188<br>n.s.	,181<br>n.s.	,202<br>p=,084
RT - Angry - inverted	**,300***<br>**p=,019**	,136<br>n.s.	,225<br>p=,062	,147<br>n.s.	,144<br>n.s.	**,262***<br>**p=,036**	,212<br>p=,074	,232<br>p=,056	**,265***<br>**p=,034**
RT - Happy - inverted	,227<br>p=,060	,155<br>n.s.	**,308***<br>**p=,017**	,151<br>n.s.	,217<br>p=,070	**,291***<br>**p=,022**	**,246***<br>**p=,046**	**,256***<br>**p=,040**	**,296***<br>**p=,020**
RT - Neutral - inverted	,212<br>p=,074	,169<br>n.s.	**,240***<br>**p=,050**	,159<br>n.s.	,155<br>n.s.	,236<br>p=,053	,206<br>p=,081	,205<br>p=,081	,230<br>p=,058

Table S3. Pearson's correlation coefficients (one-tailed) between STAI scores, mean P3 amplitudes and Reaction Times (RT) for each experimental condition across all participants (N = 48).

	STAI	P3 mean amplitude Fearful upright	P3 mean amplitude Angry upright	P3 mean amplitude Happy upright	P3 mean amplitude Neutral upright	P3 mean amplitude Fearful inverted	P3 mean amplitude Angry inverted	P3 mean amplitude Happy inverted	P3 mean amplitude Neutral inverted
STAI		-,183	-,155	-,105	-,161	-,170	-,108	-,105	-,186
		n.s.	n.s.	n.s.	n.s.	n.s.	n.s.	n.s.	n.s.
RT - Fearful - upright	,288*	-,337**	-,356**	-,371**	-,284*	-,279*	-,302*	-,354**	-,318*
	p=,023	p=,010	p=,007	p=,005	p=,025	p=,027	p=,018	p=,007	p=,014
RT - Angry - upright	,230	-,315*	-,364**	-,377**	-,318*	-,278*	-,308*	-,349**	-,340**
	p=,058	p=,015	p=,006	p=,004	p=,014	p=,028	p=,017	p=,008	p=,009
RT - Happy - upright	,221	-,233	-,276*	-,355**	-,227	-,221	-,241*	-,277*	-,244*
	p=,066	p=,056	p=,029	p=,007	p=,061	p=,066	p=,050	p=,028	p=,047
RT - Neutral - upright	,246*	-,335**	-,377**	-,377**	-,320*	-,287*	-,323*	-,372**	-,328*
	p=,046	p=,010	p=,004	p=,004	p=,013	p=,024	p=,013	p=,005	p=,011
RT - Fearful - inverted	,221	-,290*	-,352**	-,378**	-,318*	-,245*	-,336**	-,342**	-,323*
	p=,066	p=,023	p=,007	p=,004	p=,014	p=,047	p=,010	p=,009	p=,013
RT - Angry - inverted	,300*	-,358**	-,379**	-,391**	-,349**	-,326*	-,358**	-,395**	-,347**
	p=,019	p=,006	p=,004	p=,003	p=,008	p=,012	p=,006	p=,003	p=,008
RT - Happy - inverted	,227	-,380**	-,413**	-,464**	-,389**	-,327*	-,389**	-,430**	-,374**
	p=,060	p=,004	p=,002	P<,001	p=,003	p=,012	p=,003	p=,001	p=,004
RT - Neutral - inverted	,212	-,376**	-,411**	-,438**	-,376**	-,339**	-,383**	-,408**	-,389**
	p=,074	p=,004	p=,002	p=,001	p=,004	p=,009	p=,004	p=,002	p=,003
